# Wearable Metamaterial Dual-Polarized High Isolation UWB MIMO Vivaldi Antenna for 5G and Satellite Communications

**DOI:** 10.3390/mi12121559

**Published:** 2021-12-14

**Authors:** Adam R. H. Alhawari, Tale Saeidi, Abdulkarem Hussein Mohammed Almawgani, Ayman Taher Hindi, Hisham Alghamdi, Turki Alsuwian, Samer A. B. Awwad, Muhammad Ali Imran

**Affiliations:** 1 Electrical Engineering Department, College of Engineering, Najran University, Najran 66462, Saudi Arabia; ahalmawgani@nu.edu.sa (A.H.M.A.); athindi@nu.edu.sa (A.T.H.); hg@nu.edu.sa (H.A.); tmalsuwian@nu.edu.sa (T.A.); 2 Electrical and Electronic Engineering Department, Universiti Teknologi PETRONAS, Bandar Seri Iskandar 32610, Malaysia; 3 Deanship of Information and Communication Technology, Imam Abdulrahman Bin Faisal University, Dammam 31441, Saudi Arabia; saawad@iau.edu.sa; 4 James Watt School of Engineering, University of Glasgow, Glasgow G12 8QQ, UK; muhammad.imran@glasgow.ac.uk; 5 Artificial Intelligence Research Centre (AIRC), Ajman University, Ajman 20550, United Arab Emirates

**Keywords:** MIMO antenna, 5G communication, satellite communication, UWB antenna, isolation, vivaldi antenna, metamaterials

## Abstract

A low-profile Multiple Input Multiple Output (MIMO) antenna showing dual polarization, low mutual coupling, and acceptable diversity gain is presented by this paper. The antenna introduces the requirements of fifth generation (5G) and the satellite communications. A horizontally (4.8–31 GHz) and vertically polarized (7.6–37 GHz) modified antipodal Vivaldi antennas are simulated, fabricated, and integrated, and then their characteristics are examined. An ultra-wideband (UWB) at working bandwidths of 3.7–3.85 GHz and 5–40 GHz are achieved. Low mutual coupling of less than −22 dB is achieved after loading the antenna with cross-curves, staircase meander line, and integration of the metamaterial elements. The antennas are designed on a denim textile substrate with $\varepsilon_{r}$ = 1.4 and h = 0.5 mm. A conductive textile called ShieldIt is utilized as conductor with conductivity of 1.8 × 10^4^. After optimizing the proposed UWB-MIMO antenna’s characteristics, it is increased to four elements positioned at the four corners of a denim textile substrate to be employed as a UWB-MIMO antenna for handset communications, 5G, Ka and Ku band, and satellite communications (X-band). The proposed eight port UWB-MIMO antenna has a maximum gain of 10.7 dBi, 98% radiation efficiency, less than 0.01 ECC, and acceptable diversity gain. Afterwards, the eight-ports antenna performance is examined on a simulated real voxel hand and chest. Then, it is evaluated and compared on physical hand and chest of body. Evidently, the simulated and measured results show good agreement between them. The proposed UWB-MIMO antenna offers a compact and flexible design, which is suitably wearable for 5G and satellite communications applications.

## 1. Introduction

A wearable device, often known as a wearable, is a mechanism that may be worn on the body. They come in a variety of shapes and sizes, communication gadgets, augmented reality (AR) helmets, and smart watches. These gadgets can record locations, steps, beat rates heart, nearby buildings [1]. Antenna design necessitates an antenna size smaller than half the wavelength to properly collect the emitted signal. For example, consider an innovative triband antenna design that may be used in small-size wearables [2]. There are several difficulties in designing antennas when they encounter body, such as the impacts of high loss due to the high relative permittivity of human tissues which degrade the wearable antenna performance [3]. Therefore, they must be properly constructed to retain performance if they face an environment except air [4]. Moreover, wearable designs should adhere to regulations for absorption rates (SARs). Numerous two-dimensional structures such as metamaterial (MTM) structures [5,6], ferrite sheets [7,8], soft surfaces, frequency selective surfaces (FSS), and large ground planes [9,10] have been employed as insulating layers in body area network (BAN) applications to shield the human body from the harmful radiation [11,12,13].

In cellular communication, as the number of users grows, the frequency provision becomes insufficient due to limited channel capacity. Within the same frequency band, the number of operators cannot go beyond a certain limit. As the total users grow so does co-channel interference. The capability of transiting data using 3G and 4G frequency channels is restricted, streaming videos and transferring big files problematic. Larger bandwidths and quicker communication channels are required [14]. The primary spectrum bands between 2 GHz and 6 GHz are between 3.3 GHz and 4.2 GHz, and between 4.4 GHz and 4.990 GHz. These bands are now being evaluated for 5G network early testing in several countries [15]. Furthermore, several higher bands, such as 24–27.2 GHz, 33–44.13 GHz [16], 26–31 GHz [17], and 28–38 GHz [18], are also considered 5G. 5G and MIMO communication systems are two of the most significant developments in boosting bandwidth (MIMO). On the other hand, ultra-wideband (UWB) systems have numerous benefits, including a very wide bandwidth, low power consumption, a high data rate, a high temporal resolution, resistance to interference, coexistence with narrowband systems, and so on. As a result, UWB technology is widely utilized in communication, radar, imaging, and localization [19]. Multi-input-multiple-output (MIMO) technology can be used with UWB technology to increase transmission rate and communication dependability. Multiple antennas must cohabit in the limited space of transmitters and receivers [20] in order for the combinative system to function. In other words, the combination of UWB and MIMO technologies, as well as the utilization of space multipath and parallel transmission of numerous signals, may provide obvious multiplexing and diversity gain, as well as long-distance signal transmission stability [21,22,23,24,25]. In real situations, this combination can also resolve multipath fading.

When considering single antennas, features such as wide BW throughout the full working BW and improved isolation from nearby arrays come to mind. In the UWB-MIMO system, however, this is not the case. Because as the number of arrays rises, the coupling effects become more severe. This mutual coupling effect distorts antenna radiation patterns, changes antenna impedance matching, and causes correlation in sent or received signals, resulting in a decrease in MIMO system capacity [26,27]. Mutual coupling between each antenna element is unavoidable in a MIMO system, and it has a detrimental impact on the antenna’s properties. It degrades pattern correlation and radiation efficiency. In MIMO and array antennas, the decoupling approach is used to suppress mutual coupling (improve isolation) [28].

As a result, an effective strategy for decoupling should be adopted. The most basic method of decoupling is to increase antenna separation distance; however, the size of each component must be closely regulated for downsizing UWB mobile terminals. The right approach is to create a unique structure that reduces coupling without compromising transceiver space. The most frequent decoupling structures for plane monopole MIMO antennas are parasitic components and defective grounds [29,30]. Another method for creating polarization and pattern diversity in UWB-MIMO antennas is to utilize slot antennas with orthogonal feeding [31,32].

Several bands have been used for 5G communications including 3.4–3.6 GHz known as LTE and 3.4–3.8 GHz [33,34]. To satisfy the communication system’s criteria, a MIMO system must offer excellent constancy while maintaining a low HPBW value [35]. Several approaches exist for decreasing mutual coupling, involving utilization of neutralization lines [36,37], parasitic components loading [38], and decomposition methods [39]. Not all these approaches might be used efficiently in a MIMO system and cannot be appropriate for Massive MIMO systems. In contrast, metamaterial (MTM) and metasurface structures have showed promises in terms of improving mutual coupling. They are, however, constrained by the amount of design components that may be introduced [40,41,42,43]. As specified in [44,45,46,47,48], certain 5G antennas must be de-signed with small dimensions. Because the single-mode antenna components utilized to construct MIMO antennas, they had a poor data rate, a restricted BW, and a limited gain [3].

This paper is divided into four pieces. Section 1 begins with a detailed overview of current articles on MIMO antennas, their technology, and the problems associated with 5G communication systems. Section 2 shows the suggested MIMO antenna and design processes of metamaterial, as well as their performance evaluation. After evaluating the antenna’s ability to operate as a high isolation and efficiency MIMO antenna, four elements combined on an individual textile to be considered as a wearable eight-ports MIMO antenna. Section 3 presents the findings of this investigation. Finally, Section 4 contains the final observations.

## 2. Configuration and Design of the Proposed UWB-MIMO Antenna

The fundamental properties of a suggested prototype comprising a modified antipodal Vivaldi (MAVA) patch integrated with ten MTM components are studied, and its capacity to function as a UWB-MIMO transceiver element for 5G and satellite communications is demonstrated. First, a modified antipodal Vivaldi antenna was developed, and its properties were evaluated. The antenna was then loaded with arcs linked to the patch around the intersection of the transmission line and patch to improve the BW and remove the stop band near the X-band (frequency band of 7.25–11 GHz). They were combined. Following that, the antenna was loaded with a number of staircase meander lines. Following that, a cell of MTM was created and characterized at 5G and satellite bands. The MTM components were then combined to enhance the UWB-MIMO antenna radiation qualities by reducing mutual coupling, increasing isolation, and increasing diversity gain, as well as improving the radiation characteristics.

### 2.1. MAVA

**Figure 1:**
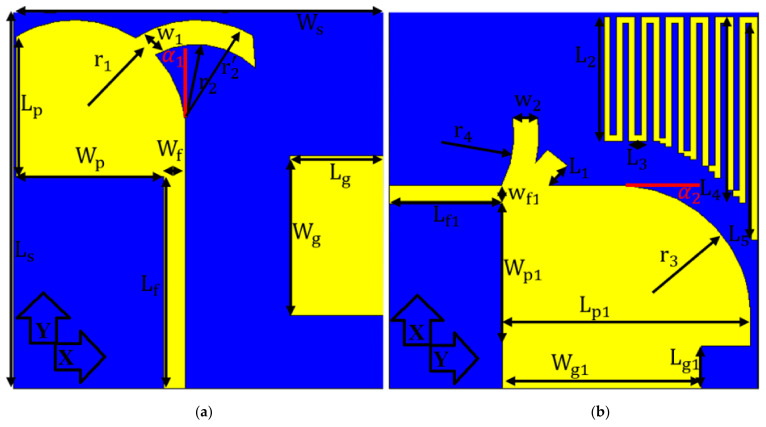
(**a**) front and (**b**) back view of the simulated prototype of the proposed antenna.

The MAVA has a modified antipodal Vivaldi patch shape on both sides of the denim substrate to attain dual polarization and an improved directional pattern. Figure 1 and Table 1 show the MAVA antenna’s structure and dimensions. The ShieldIt conductive textile of the denim substrate has a $\varepsilon_{r}$ = 1.4, a loss tangent of tan δ = 0.02, and a h = 0.5 mm (thickness). Vivaldi antennas are typically low-frequency resonance structures. The resonance frequency varies with width at lower frequency. The substrate thickness is another element that might affect the performance of the antenna. When it is too huge, the undesirable modes are activated. The pattern is distorted, and the level of cross-polarization is enhanced when the modes that are not wanted. Thus, a low dielectric constant substrate and a modest thickness is desired. The length of the antipodal patch is also effective for antenna’s performance improvement. Consequently, it can affect the gain, the lower and higher resonances [49,50].

Antipodal Vivaldi Antennas (AVAs) have been suggested and deployed in a variety of applications to enhance the BW [51]. The feed line and tapered slot transition are precisely specified to prevent BW and gain decrement when compared to a traditional Vivaldi antenna. Furthermore, unlike traditional Vivaldi antennas, AVAs have minimal cross-polarization and may change the radiation beam [52]. As a result, the research focus is on increasing AVA performance. For example, the authors of [53] proposed using a dielectric lens to concentrate energy. Another paper [54] advocated the use of synthetic materials, while [55] proposed the use of a parasitic elliptical patch. This study was inspired by AVA and then enhanced with a low-profile and robust gain, as well as a better feeding technique for 5G and X-band communications. An exponential feed line, a stairwell meander line, and metamaterial (MTM) loadings are among the changes. The FIT analysis in Computer Simulation Technology (CST) Microwave Studio was utilized to examine the antenna.

Figure 1 illustrates the suggested modified AVA model (MAVA), which is selected because of its favorable performing. Initially, a typical AVA was modified, simulated, and developed at 5–40 GHz, and next the dimensions were optimized. Particularly, exponential patch and feed line dimensions were calculated applying the formulas provided in [56,57]. The proposed antenna includes two main tapered strips (conventional structure) and three additional curved-strip arms. It will be investigated how these additional curved-strip arms contribute to convert stopbands to passbands. In order to have a compact UWB antenna, four exponential curves (two as patch and three curved strips) are employed. The conventional equations of these curves can be given as (Equation (1) for the patches and Equation (2) for curved strips):

(1) $$\left\{\begin{array}{c} y\left(x\right)=A_{1}e^{A_{2}x}+A_{3}\\ y\left(x\right)=B_{1}e^{B_{2}x}+B_{3} \end{array}\right.$$

(2) $$y\left(x\right)=C_{1}e^{C_{2}x}+C_{3}$$

(3) $$y\left(x\right)=\pi \;\left(r_{2}^{\prime 2}-r_{2}^{2}\right)$$

where *A*_2_, *B*_2_, and *C*_2_ demonstrate the degree of the exponential curves and *A*_1_, *B*_1_, *C*_1_, *A*_3_, *B*_3_, and *C*_3_, are related to the length and width of the arms and depend on the position of the origin of the Cartesian coordinate system in the antenna structure. To have the optimum reflection coefficient, the values of *A*_2_, *B*_2_, and *C*_2_, should be optimized to get the best matching. Furthermore, $r_{2}^{\prime }$ and *r*_2_ are the internal and external radiuses of the curved strip shown in Figure 1.

To solve these equations, for instance for the curved stub, the input impedance for a stub loaded microstrip patch is calculated by the general planar dielectric dyadic Green’s function approach in the spectral domain, as was initiated much earlier and has been extensively expanded upon and utilized successfully throughout the literature for microstrip antenna configurations. Using the spectral domain dyadic Green’s function derived earlier [58] with the electric field integral equation, the problem is formulated by using entire domain basis functions to represent the surface current densities on the patch, the loading stub and the attachment mode at the junction. Galerkin’s procedure can be used to reduce the electric field integral equation to a matrix equation, which is then solved to obtain the amplitudes of the surface currents. These surface currents are then used for calculating the input impedance of stub loaded rectangular and circular microstrip patches. Numerical results are compared with measured results and with previous results calculated by the Thevenin’s theorem approach. More details about the equations and explanation are presented in [59].

The radiation region is formed by two MAVA patches. The patch and feed line dimensions of the first antenna are L_p1_, L_f1_, W_f1_, W_p1_, W_g1_, and L_g1_, as illustrated in Figure 1. Equations (4) and (5) were used to calculate the actual size of the exponential ground section (the points location is shown by *x*).

(4) $$Y_{1}\left(x\right)=-\left(0.25e^{\frac{x-2}{2}}+6.5\right)$$

(5) $$Y_{2}\left(x\right)=-\left(0.24^{\frac{x-1}{2}}+5.8\right)$$

This shape is utilized to reduce mutual coupling, heterogeneous tapering, create capacitive spaces for reduction of cross-polarization, and produce an input impedance of 50 ohm. Then, at the intersection of the feed line and patch, two cross curves with lengths of L_1_, W_2_, and radius of r_4_ were added to produce a pass band at 5.2 GHz. L_p_, L_f_, W_f_, W_p_, W_g_, and L_g_ are the second antenna’s effective parameters. Their optimal values were displayed in Table 1. Another MAVA-like antenna was constructed on the other side of the substrate. It is fed through a length and breadth of L_f_ and W_f_, respectively, of an exponential transmission line (an arrangement of an up-front feed line and a quarter of circular disc with a radius of *r*_1_ = 4.15 mm). 

After optimizing both antennas to gain the mandatory working bands, they were combined to produce an antenna with two operational modes (Ant 1: Figure 6a) and (Ant 2: Figure 6b) able of functioning both horizontally and vertically polarization UWB-MIMO antenna for lower and higher 5G, Ka and Ku bands, and X-band communication applications.

**Figure 2:**
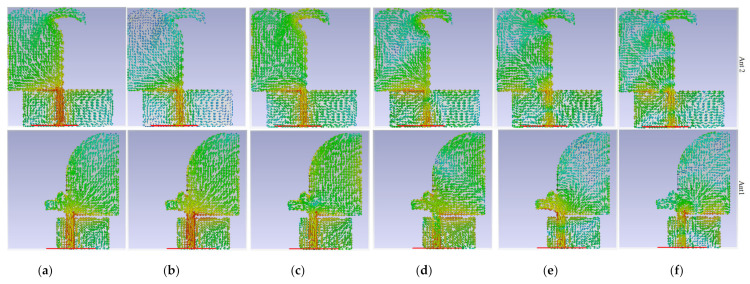
The SCD for Ant 1 and Ant 2 separately, before they were combined: (**a**) 3.8 GHz, (**b**) 5.2 GHz; (**c**) 8 GHz, (**d**) 15 GHz, (**e**) 22 GHz, and (**f**) 28 GHz.

Adding the curve with radius r_2_ and width W_1_ added a new resonance frequency and decreased the fringing field, although also somewhat moved the band to the higher band. Around 15 GHz, that similarly enhanced impedance matching. To decrease the degrading radiations and improve the antenna’s radiation efficiency, the patch in the first antenna is cut adjacent at the edges of the substrate (the cut length is L_g1_). By examining the surface current distribution at the required frequencies, the gaps impact, the tapered angle, and the optimum sizes were studied. Knowing how to convert a stop band to a passband is also beneficial. Figure 2 depicts the surface current density (SCD) in targeted working resonances (3.8 GHz, 5.2 GHz, 8 GHz, 15 GHz, 22 GHz, and 28 GHz). At different frequencies, the amount of the SCD on the ground close to the patch on the other side, the transmission line, and the two cross-curves are the strongest for Ant 1. Furthermore, these two cross-curves produce and distribute current to inhibit surface waves and coupling that may accumulate in that area, primarily at 3.8 GHz, 5.2 GHz, and 15 GHz, correspondingly. At 3.8 GHz, 5.2 GHz, and 22 GHz, Ant 2 shows the strongest current near the transmission line, the ground, and the curve linked to the patch. Additionally, Figure 2 indicates that the SCD for Ant 2 is stronger over the curve at 8 GHz, the exponential section of the patch at 22 GHz, and it exhibits greater density around the patch through the cross-curves at 22 GHz for Ant 1.

**Figure 3:**
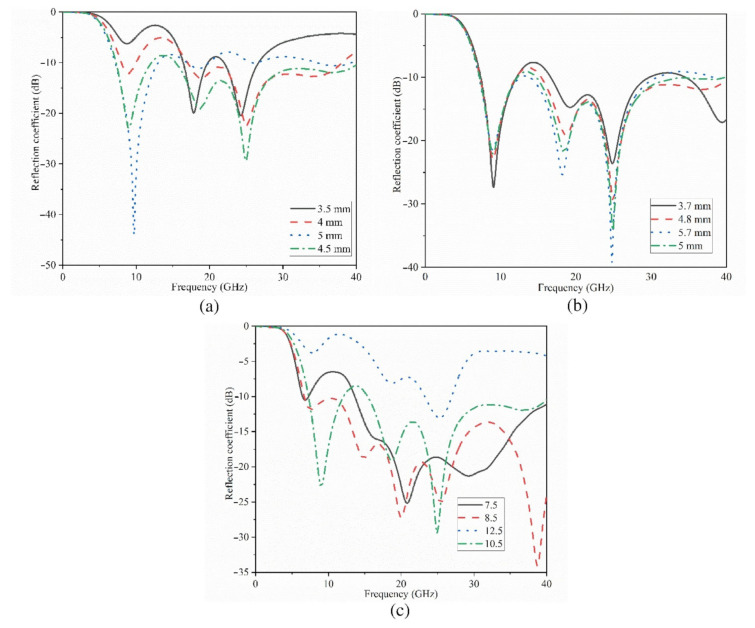
Parametric study of the Ant1: (**a**) feed line (L_f1_), (**b**) ground length (L_g1_), and (**c**) ground width (W_g1_).

The findings of a parametric analysis of Ant 1’s essential characteristics in terms of ground dimensions (L_f1_, W_g1_) (Figure 3b,c) and exponential transmission line length (L_f1_) are shown in Figure 3. (Figure 3a). Figure 3b,c show that the ground length and breadth of Ant 1 influence the resonance across the full working BW. However, the more ground length is increased, the higher frequency range slightly shifts to a lower frequency region. Yet, the level of the reflection coefficient has not changed significantly.

In the case of stopband, it occurred at 15–18 GHz for ground lengths smaller than 4.8 mm. In particular, the circumstance is considerably different in terms of ground breadth, which the optimized parameters are between 8–9 mm, e.g., if the working BW was increased higher than 11 mm, it would be completely disrupted. Figure 3a depicts the change of transmission line length (L_f1_). This graph demonstrates that increasing this length has minimal effect on the lower resonance frequency, but it moves the band to some extent. Furthermore, an increase in this length has the greatest impact on the working BW in terms of impedance matching and reflection coefficient level. It should be noted that the line width enhances the matching when the substrate thickness is considered (the microstrip design principles and equations should be followed). 

**Figure 4:**
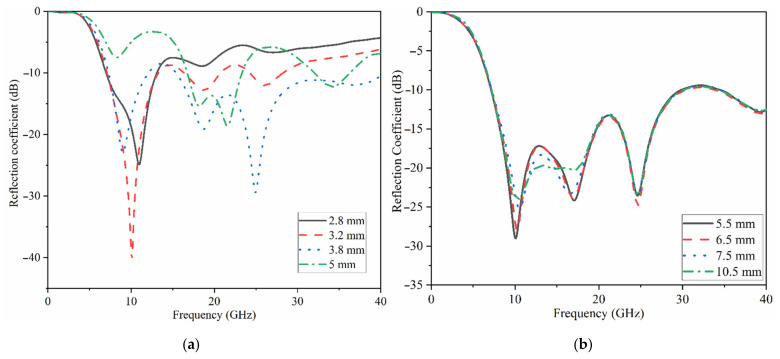
Parametric study of Ant 2: (**a**) ground length, L_g_ and (**b**) the patch’s length, L_p_.

**Figure 5:**
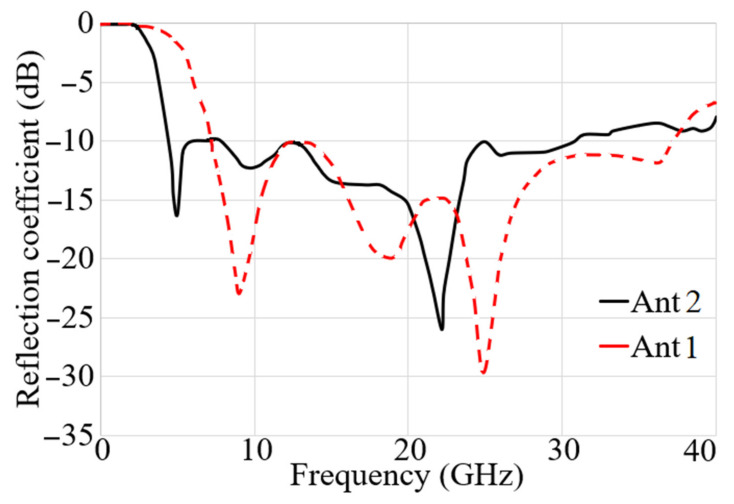
S_11_ results of both antennas.

The changes in the Ant 2’s reflection coefficient in terms of ground length (L_g_) and patch length (L_p_) dimensions are depicted in Figure 4a,b, correspondingly. The outcomes reveal that when the ground length is less than 3.5 mm (L_g_ < 3.5 mm), most of the higher frequency band is disrupted and vanishes, and the lower band is moved to the higher frequency band as well (this is due to the fact that the ground length play an important role in impedance matching of UWB antennas). Its width, on the other hand, influences the impedance BW and aids in its improvement. For example, increasing the width of the working BW until it touches the region where the bordering fields have a detrimental effect. Figure 4b shows the patch length sweeps. As seen, increasing the patch length increases the resonance frequency and somewhat shifts it to the low resonances. The patch width, on the other hand, influences the bandwidth over the entire band since an increase in width reduces the resonance and therefore the working BW. The reflection coefficient results for both antennas when the sizes are optimum are shown in Figure 5. The achieved working bandwidth of 3.4–31 GHz and 7–37.5 GHz for antenna 2 and antenna 1 achieved, respectively. 

**Figure 6:**
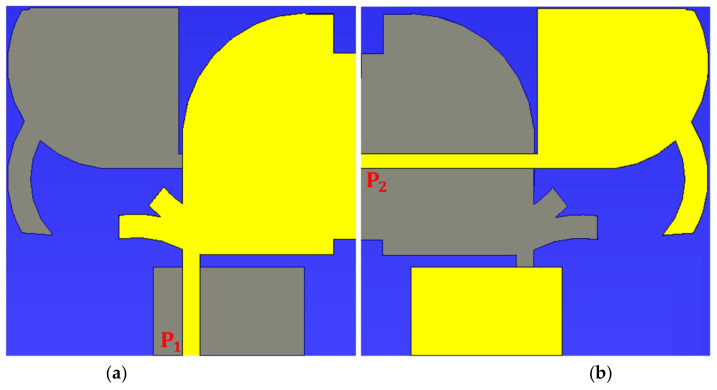
The antenna prototype after combination (**a**) front and (**b**) back views.

Both antennas were combined to form the proposed UWB-MIMO with both vertical and horizontally polarization antenna, which would be used in a communication system for lower and higher 5G, X-band, Ka and Ku, and satellite communications. Figure 6 depicts the proposed antenna’s 2D views following integration with.

**Figure 7:**
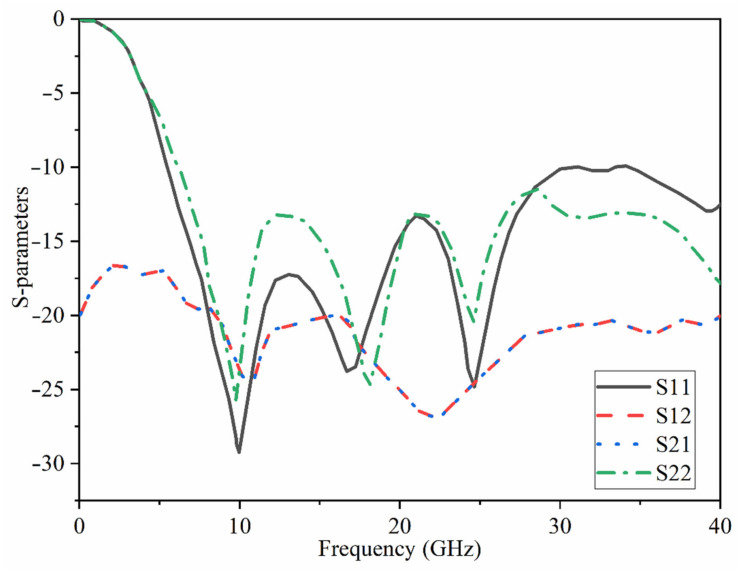
The antenna’s simulated reflection and transmission coefficients results.

Ant 1 and Ant 2 modes of operation give vertical and horizontal polarizations, respectively. W_f_ and W_f1_ are taken into account to match the width of the feed lines. L_f_ and L_f1_ also have an impact on the matching. We included SMA connections in the simulations to account for the influence of the connectors (two different ports used here (only normal SMA which covers till 18 GHz is shown). After we measured the antenna and were sure that the results agreed, the ports changed to the new one taken from the following port. [60]). High isolation is expected because two radiation modes (Ant 1 and Ant 2) are both horizontally and vertically polarized. The simulated S_11_ and S_21_ results of the combined antennas are presented in Figure 7. The high level of isolation is evident since the S_21_ value is less than −18 dB. In addition, a perfect impedance matching was also obtained when reflection coefficients ≤−10 dB. The antenna demonstrates the matching at most of the band for both antennas’ operational modes.

A staircase meander line near the Ant 1 parasitically loads the planned UWB-MIMO antenna. This meander line’s staircase nature enhances the BW shifting it to the lower band like here, it yields a resonance at 3.8 GHz, which covers the lower 5G spectrum too. The reflection coefficient results with the staircase meander line are shown subsequently, together with its results for each step of antenna design and loading [61,62,63]. 

**Figure 8:**
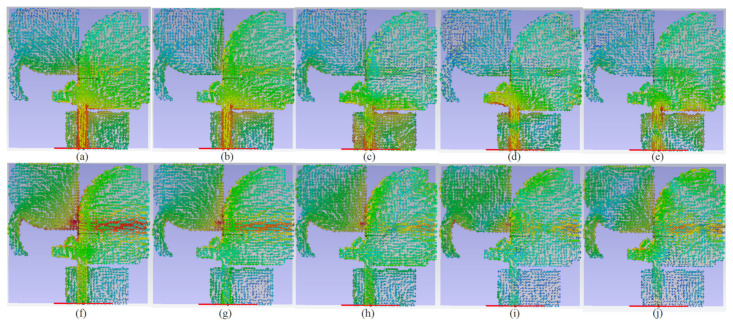
Distribution of the SCD after Ant 1 and Ant 2 were combined at: (**a**,**f**) 3.8 GHz, (**b**,**g**) 5.4 GHz, (**c**,**h**) 8 GHz, (**d**,**i**) 15 GHz), and (**e**,**j**) 28 GHz.

SCD of the two modes at operating BW is depicted in Figure 8. The planned antenna’s SCD at the lower and upper ends of the BW, as well as the poles in the operating BW for the two ports, were examined to assure dual-polarization functioning. Figure 8a–e is the SCD when port 1 is active and Figure 8f–j when port 2 is active. Figure 8 illustrates that while port 1 is active, the SCD travels along the y-axis, but when port 2 operates, SCD flows in the direction of the x-axis. 

**Figure 9:**
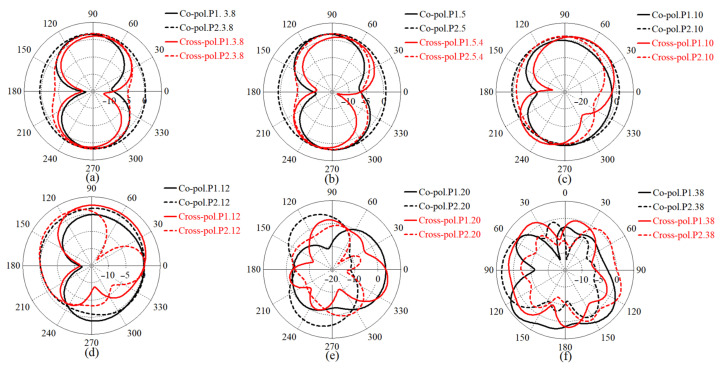
The radiation patterns of MAVA antenna considering both Ant 1 and Ant 2 at: (**a**) 3.8 GHz, (**b**) 5.4 GHz, (**c**) 10 GHz, (**d**) 12 GHz, (**e**) 20 GHz, and (**f**) 38 GHz before combination and loadings.

The polarization diversity is one of the most important antenna diversity techniques that helps in improving the signal quality as well as enhancing the channel capacity and spectrum efficiency [64]. In general, Co-polarization is the desired polarization of the wave to be radiated by the antenna. On the contrary, cross-polarization (X-pol) is the orthogonal radiation of the desired polarization of wave (the polarization of above wave). For instance, if the desired polarization is horizontal, the X-pol is vertical. Figure 9 depicts the 2D radiation patterns of the proposed antenna for the antenna modes at working BW, lower end, and upper end of the working BW before they were combined and the loadings (meander line and MTM structure) were added. At the resonance frequencies, the simulated radiation pattern for Ant 1 (towards co-polarization) and Ant 2 (towards co-polarization) does not change much from the measured radiation pattern.

It can be noticed that the ground and the patch are connected. It is to improve isolation, thus ensuring a common reference voltage level in the ground plane of the MIMO antenna. However, it is quite difficult to retain circular polarization behavior of the antenna elements in the ground radiating CP MIMO structures when the ground patches are connected together. This is due to the fact that the connecting strip is made up of conductor, and, therefore, it will leak some amount of current toward the neighboring antenna elements [65]. To avoid this effect the antenna is thus loaded with MTM structure.

### 2.2. MTM Elements and Array Integrations 

Periodic structures are used to create metamaterials (MTM). They are used to obtain a broad bandwidth and greater directional gain while keeping a low-profile construction. In the direction of end-fire radiation, we suggest employing MTM in a planar structure.

**Figure 10:**
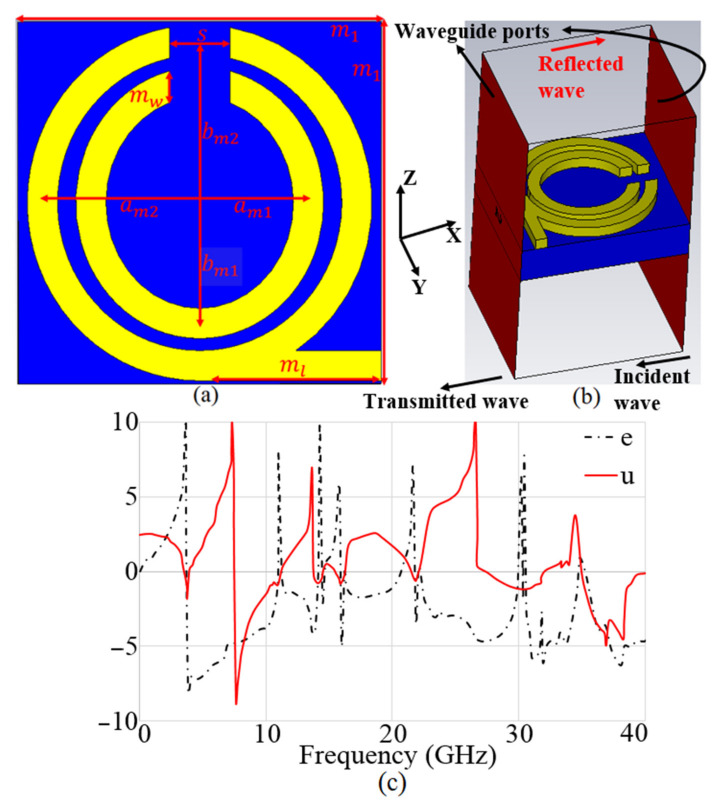
MTM unit cell structure: (**a**) 2D view, (**b**) 3D view and (**c**) effective permittivity (*e*) and permeability (*u*).

The suggested MTM unit cell is illustrated in Figure 10a. The primary model of the MTM unit cell is based on a Split-Ring Resonator (SRR) structure with two circular rings [66]. The SRR is modelled as a magnetically resonator affected by a vertical magnetic field to create permeability [67]. Splits (gaps) are usually carved out of structures to produce capacitance and check the structure’s resonant specification. The MTM structure is tuned to function in the necessary frequency spectrum by adding a Capacitive-Loaded Strip (CLS). It should be noted that a CLS functions in the same way as an electric dipole [68]. The proposed MTM shape is only present on one side of the substrate. 

**Figure 11:**
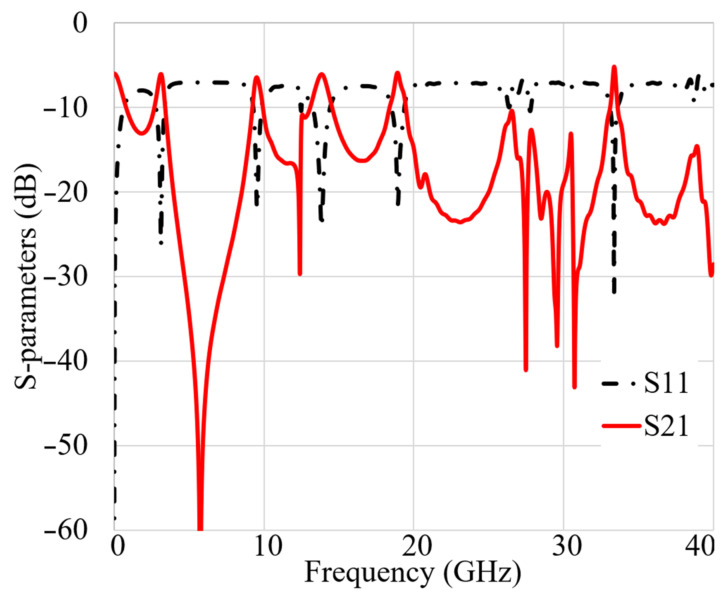
MTM unit cell structure S-parameters.

**Figure 12:**
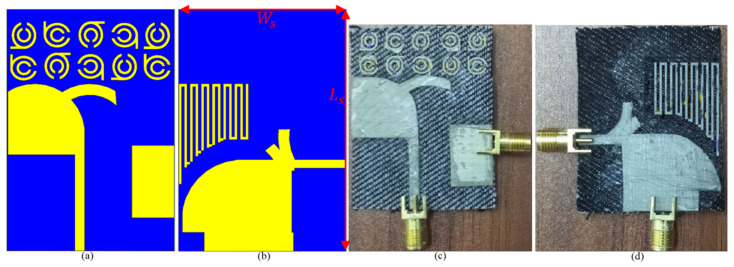
The final MAVA combined with the MTM: (**a**,**b**) the simulated structure, (**c**,**d**) the fabricated prototype ($L_{s}=21.5$ mm, $W_{s}$ = 15 mm).

Figure 10a,b displays the unit cell of MTM on a denim substrate with a $\varepsilon_{r}$ = 1.4, tan δ = 0.02, and h = 0.5 mm thickness. Perfect magnetic and electrical barriers surround the MTM structure (xz and yz planes, respectively). Parallel to the xy planes, two waveguide ports are attached to the two sides of the MTM cell. In the direction of the z-axis, an open border is assumed. Utilizing the simulated S_11_ and S_21_, the Nicolson–Ross–Weir (NRW) technique [69] was used to evaluate the electromagnetic properties of the proposed MTM unit cell in order to ascertain and then extract the $\varepsilon_{r}$ and $\mu_{r}$ as in (Figure 10c). Figure 10 summarizes the operational BW range of $\varepsilon_{r}$ and $\mu_{r}$. After that, ten units of the suggested MTM were added to the antenna to enhance its characteristics in terms of gain, directivity, radiation efficiency, and impedance matching by suppressing surface waves caused by the reduced tapering angle. Figure 11 indicates the reflection and transmission coefficient results for the single cell of MTM structure. Figure 12 shows how the MTM components were rotated in different orientations at every 90° increments.

In order to improve the performance antenna while maintaining a compact printing area, the angle (α) was lowered to generate greater focus towards the broadside direction. That will minimize the antenna’s breadth after the ideal dimensions of the proposed MAVA antenna were determined. Consequently, the decrement affected the surface waves increment, thus, the antenna’s performance as well.

### 2.3. Modeling and Characterization of the MAVA Combined with MTM Cells

The findings of the proposed UWB-MIMO antenna’s integration with ten components of a modified SRR MTM are provided in this section. The optimum dimensions and properties of the MTM structure are shown in Table 2 and Table 3. Figure 12 shows the proposed antenna integrated with MTM components in both simulated and manufactured prototypes, as well as the measurement setup for the constructed antenna. Each unit cell was added with space of almost 4 mm which is less than a quarter wavelength at the center frequency. 

The MTM arrays are located without any disorientation initially. Then they were periodically rotated by 90 degrees. This was inspired by the fact mentioned above. It was also inspired by the quasi-random solution was proposed in [70] to improve the performance of random metamaterial structures. Random metamaterial structures could potentially be used as RF wave filters or frequency selective surfaces (FSS) for specific frequency-dependent applications. This was done under the hypothesis that performance of each local random element may improve if each element is perpendicular to the incident wave, such as in many periodic metamaterial simulations. It was used to improve the radiation characteristics of antennas like gain after integration depend on their interactions. Moreover, it is also helpful structure if an experiment needed a signal transmitted or received with random fluctuations in the pattern based on direction [70].

The antenna’s overall size increased by 6.5 mm in the y direction after the MTM components were added. Next, the proposed antenna is loaded with a staircase meander line near to the Ant 1 to improve the BW and shift it to lower band as well as could create a resonance at 3.8 GHz. So that means the lower 5G band coverage is achievable, too [71,72,73,74].

**Figure 13:**
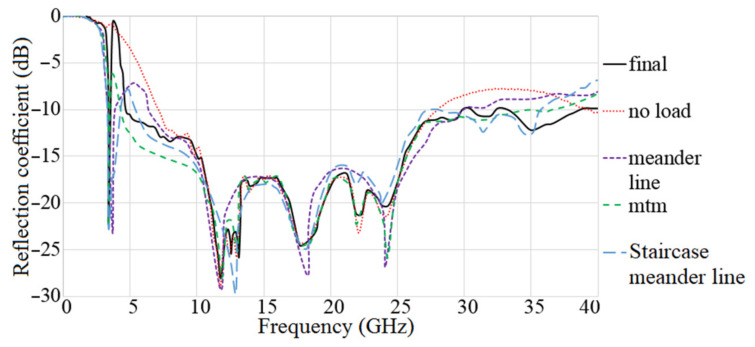
The S_11_ results of MTM based MAVA for each stage.

**Figure 14:**
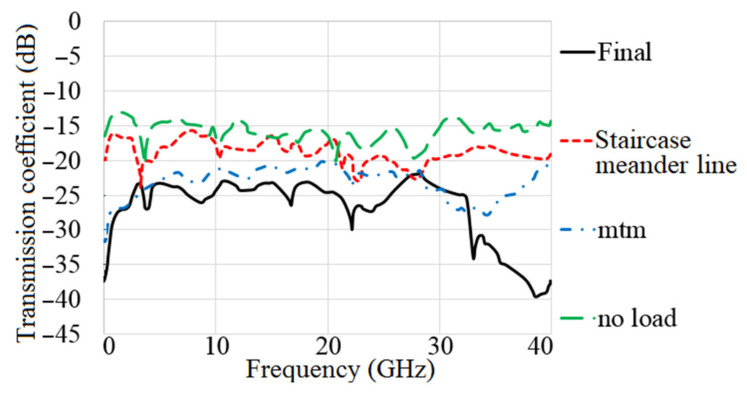
The transmission coefficient results of MTM-based MAVA at each stage.

The MAVA antenna’s simulated reflection coefficient and transmission coefficient findings at each step of the design are shown in Figure 13 and Figure 14. The researchers found that the final design of the antenna has a working BW of 4.9–40 GHz and a resonance of 3.8 GHz. Additionally, at the full working BW, the simulated transmission coefficient is less than −22 dB.

**Figure 15:**
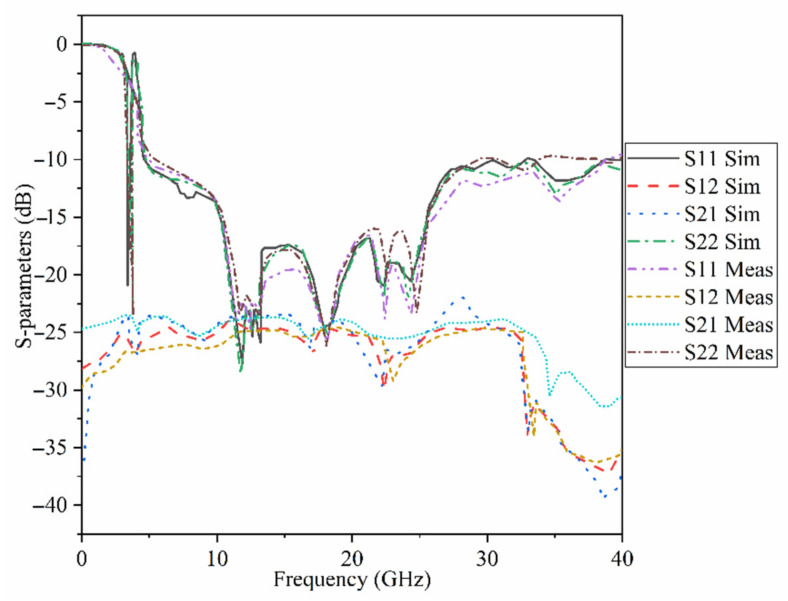
The simulated and measured S-parameters outcomes of the proposed antenna in free space (Sim: Simulation, Meas: Measurement).

Figure 15 indicates the proposed MAVA’s simulated and measured S_11_ and S_21_. A Performance Network analyzer: PNA type HP 85070-Ds was used to complete the test. The PNA was calibrated in the 100 MHz–40 GHz frequency band. The S-parameters results do not show substantial variation in Figure 15. These findings confirmed that the suggested antenna has a broad BW that meets the requirements of 5G (lower and higher bands), X-band, Ka and Ku, and satellite communication. Furthermore, there is sufficient isolation between each port (S_21_ less than −18 dB).

**Figure 16:**
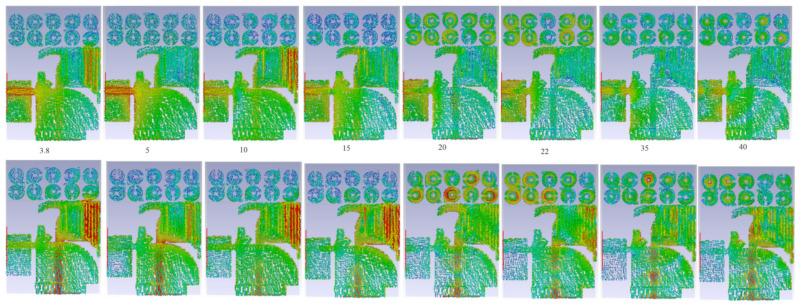
The SCD distribution of MTM-based MAVA at different frequencies: first row is for Ant1, and second row is Ant 2.

Figure 16 shows the proposed antenna’s simulated SCD after MTM has been implemented, while Ant 1 (port 1) or Ant 2 (port 2) is operational at different bands.

**Figure 17:**
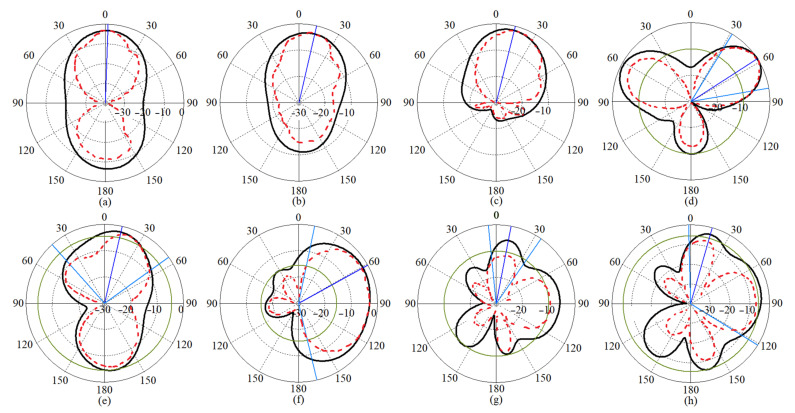
Radiation patterns of MTM-based MAVA for port 2 at: (**a**) 3.8 GHz, (**b**) 5.4 GHz, (**c**) 8 GHz, (**d**) 12 GHz, (**e**) 18 GHz, (**f**) 22 GHz, (**g**) 25 GHz, and (**h**) 28 GHz in free space (black line: simulated co-polarization, red dashed line: measured co-polarization, on the xz plane).

The antenna radiation patterns (co-polarization on the *x-z* plane) generated and measured for antenna 2 at various resonance frequencies are shown (Figure 17). Furthermore, for each mode of emission, the MTM-based MAVA had lower back radiations and side lobes than the original antenna.

## 3. The UWB-MIMO MTM-Based MAVA for Diversity Analysis 

**Figure 18:**
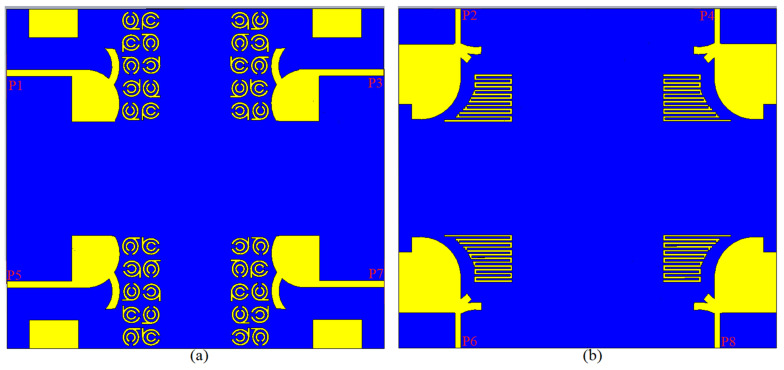
The MIMO antenna on a denim flexible board: (**a**) 2D front view, (**b**) back view.

**Figure 19:**
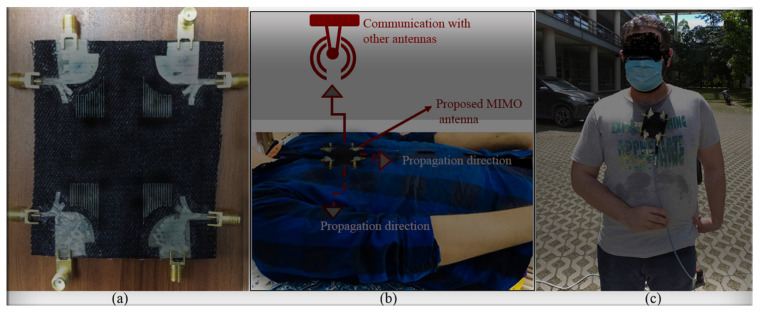
The MIMO antenna on a denim flexible board: (**a**) fabricated, (**b**) measurement setup on chest while laying down, and (**c**) measurement setup on chest standing in free space.

**Figure 20:**
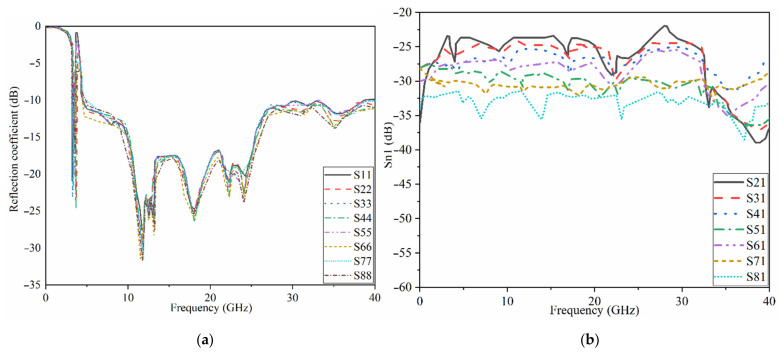
Simulated frequency response results of the proposed MIMO antenna for a mobile handset communications co: (**a**) reflection and (**b**) transmission.

The proposed MAVA were positioned at the edges of a flexible denim comparable for 5G communication applications having a total size of 50 × 45 mm^2^ (width × length), as shown in Figure 18a,b, to produce a MIMO configuration using the suggested antenna. Figure 19a,c,d shows the constructed flexible MIMO antenna and its measurement setup in the free space. The S-parameter of the flexible eight-ports is measured by connecting port 1 to PNA terminal 1 and the remaining ports to PNA terminal 2 (P_1_ sends and P_2_–P_8_ receive). Figure 20a shows the simulated S_nn_ (n is the number of ports) for an eight-port MIMO antenna. In Figure 20b, the S_21_–S_28_ for the antennas are presented. Even in the worst situation, which occurred between ports 1 and 2, the transmission coefficient data reveal a level of less than −22 dB. Within the whole frequency band, the suggested MTM-based antenna has a radiation efficiency of about 89 percent. It’s worth noting that MTM array elements are utilized to improve the suggested antenna’s maximum gain and efficiency, which are 10.7 dBi and 98 percent, correspondingly. The outcomes from the other ports are about equivalent to an individual antenna having modes of antennas. It will be informative knowing that since the ShiledIt is very sensitive towards hot soldering, we used conductive glue carefully. Because if the conductive glue used a lot around the port, the resistivity will be increased which degrades the antenna performance.

Table 4 shows the gain calculated when port 1 and 2 are active, port 1 sends and port 2 receives. However, Figure 20 shows the reflection and transmission coefficients when port 1 sends and receives. The reflection and transmission coefficients are slightly different in working BW and poles, and their levels are also different due to the mutual coupling effects between each two ports. The difference in gain and efficiency is related to the loading that was used. For instance, the meander line was loaded to enhance the BW at lower band and improve the matching there. Therefore, the radiation characteristics of the antenna is higher there.

**Figure 21:**
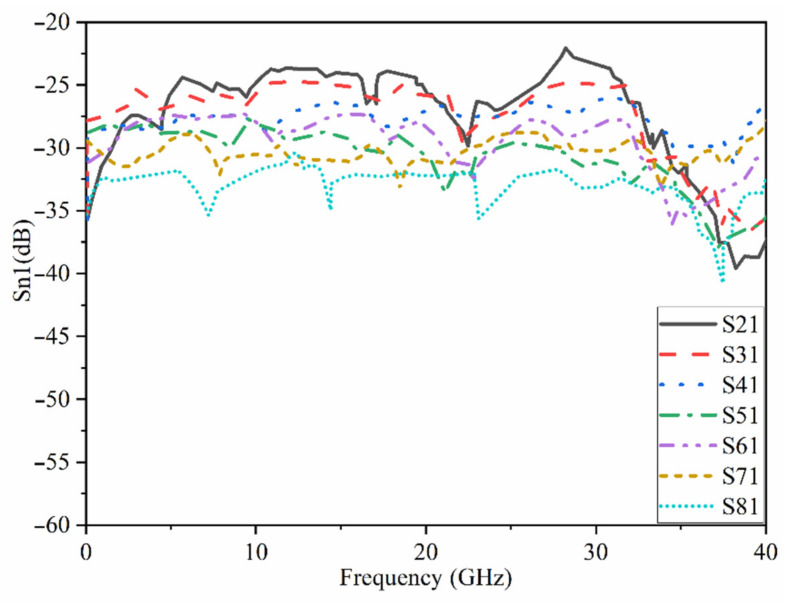
Measured transmission coefficients of the UWB-MIMO antenna.

The transmission coefficient experimental findings are shown in Figure 21. It shows that the MIMO antenna is effective over the full BW. Besides, there is strong isolation between the ports as indicated by the transmission coefficients of ports 1–8. The simulated and the measured results of the transmission coefficient yield the same pattern.

One of the most important elements in evaluating the effectiveness of a MIMO antenna system is the Envelope Correlation Coefficient (ECC). It’s used to figure out how comparable the antenna elements in a MIMO system behave and how much diversity there is between them. When ECC is less than 0.5 [71,72], it might be considered acceptable. When ECC is low, there is a lot of isolation between each antenna pair. ECC may be calculated using the antenna’s radiation pattern, as illustrated in Equation (6):

(6) ρmn=∫∫04π[Fm→θ,∅×Fn→θ,∅]dΩ2∫∫04πFm→θ,∅2dΩ∫∫04πFn→θ,∅2dΩ

where $\rho_{mn}$ is the value of ECC between the m and n ports, $\vec{F_{m}}\left(\theta ,\emptyset \right)$ is the radiation patterns of the antenna. In this work, n and $m\epsilon \left\{1,2,\cdots ,8\right\}$.

The diversity gain (DG), on the other hand, is the process of selecting the strongest signal out of a set of N signals. The Equation (7) is used to determine it [73].

(7) $$DG=10\sqrt{1-{\left(ECC\right)}^{2}}$$

**Figure 22:**
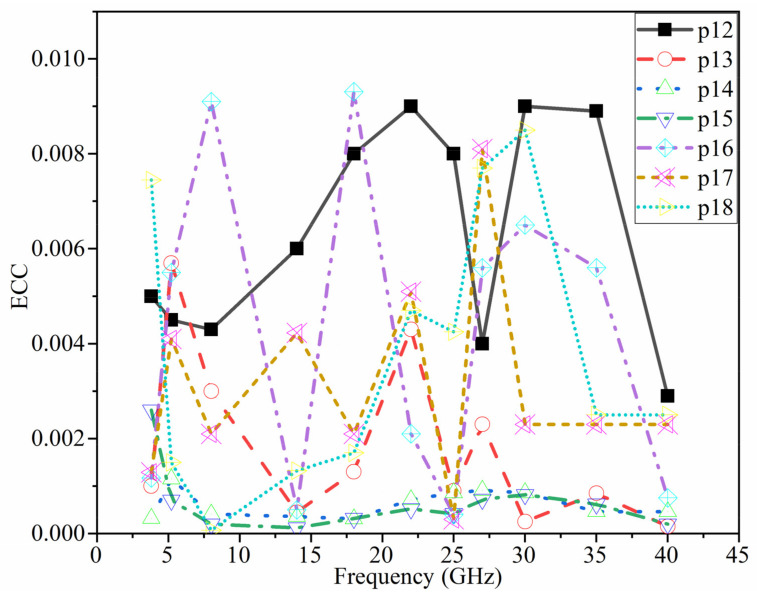
ECC alteration between port 1 and other seven ports (e.g., P_12_: ECC between Port 1 and Port 2).

Figure 22 shows the ECC values of the MIMO antenna elements when using the 3D radiation of each element. It should be noted that in each situation, a pair of MIMO components is taken into account. Because of the orthogonality of the polarization between the two neighboring antennas, very minimal ECC values are obtained, as predicted. The ECC ≤ 0.01 demonstrate that the eight-ports MIMO antenna offers strong diversity performance. (The ports shown in Figure 22 were chosen as samples) (Table 5).

Since the ECC value is $\left|\rho \right|\leq 0.3$, it can be assumed adequate to produce high diversity gain in a MIMO antenna array system.

One of the most significant diversity performance assessment metrics for MIMO antennas is channel capacity loss (CCL). It refers to the highest possible information transmission rate at which the signal may be easily transmitted without substantial loss. The following Equation (8) [73] can be used to determine the CCL.

(8) $$\mathrm{CCL}=-log_{2}\det\left(A\right)$$

where,

$$A=\left[\begin{array}{ccc} a_{11} & \cdots & a_{18}\\ \vdots & \ddots & \vdots \\ a_{81} & \cdots & a_{88} \end{array}\right],\;a_{ii}=1-\left(\sum_{j=1}^{M}{\left|Sij\right|}^{2}\right),\;a_{ij}=-\left|S_{ii}*S_{ij}+S_{ji}*S_{jj}\right|.$$

**Figure 23:**
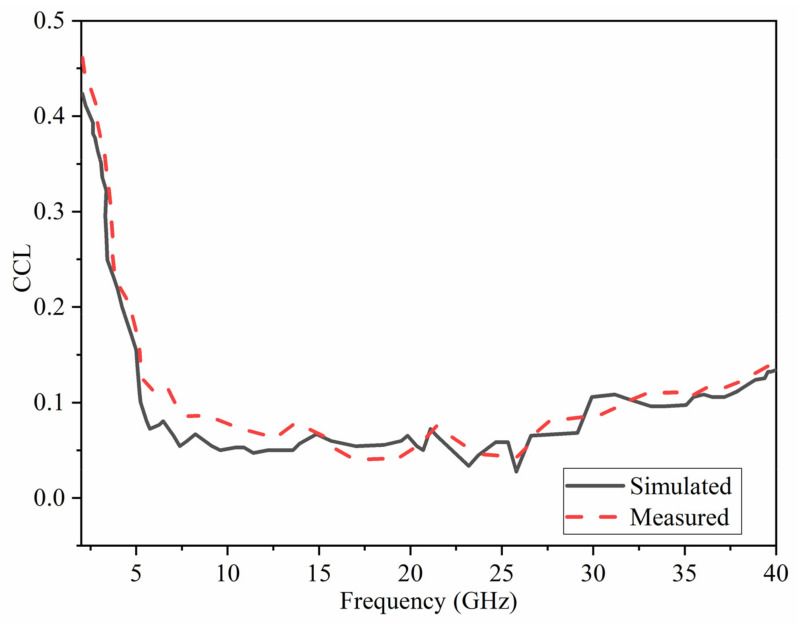
Simulated and Measured performance for the channel capacity loss (CCL).

Figure 23 depicts the CCL calculated for the proposed antenna. At most frequency bands, the estimated CCL is almost half of the typical value of 0.4 bits/s/Hz for a realistic MIMO antenna system.

**Figure 24:**
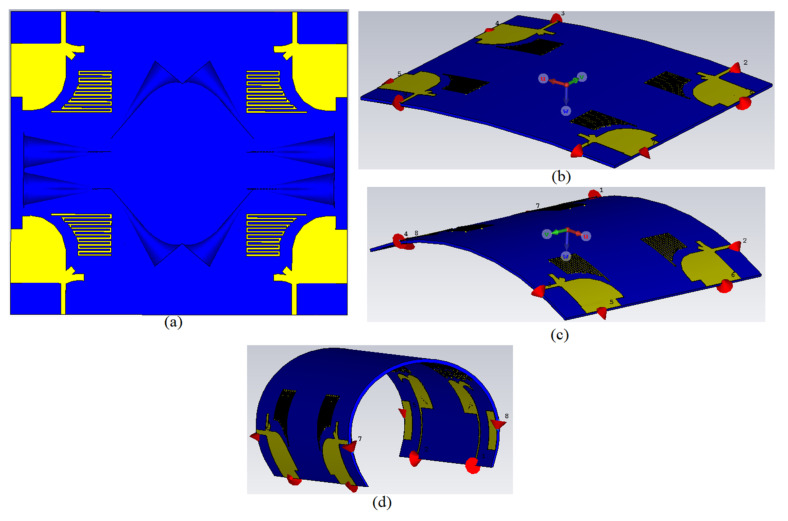
Flexibility investigation of the proposed 8 ports MIMO (**a**): creased substrate, (**b**): 10 degrees, (**c**): 50 degrees, (**d**): 190 degrees.

**Figure 25:**
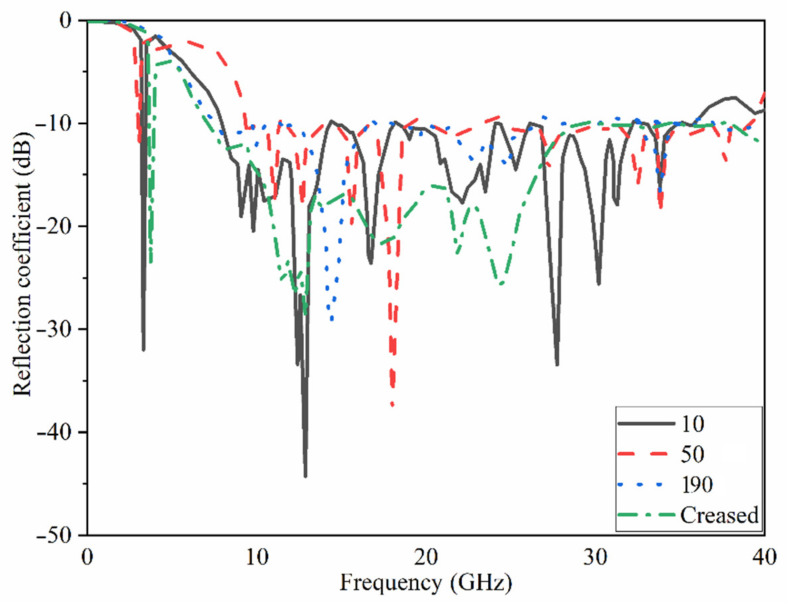
Reflection coefficient results of flexibility investigation for the proposed 8 ports UWB-MIMO (in degrees) when antenna 1 sends.

Figure 24 and Figure 25 depict the flexibility capability setup and the reflection coefficient result at various bending angles. It demonstrates that increasing the bending degree reduces the BW. Still, the decrease is minor, and the majority of the BW remains. Furthermore, when the degree increased, the amount of the reflection coefficient decreased. Apart from that, the creased substrate had no effect on the reflection coefficient findings. The bending conditions should be investigated since the proposed MIMO antenna is wearable. Furthermore, the robustness of a wearable antenna should be checked to be sure if the antenna’s performance in terms of S-parameters and other radiation characteristics are not dramatically altered [75].

**Figure 26:**
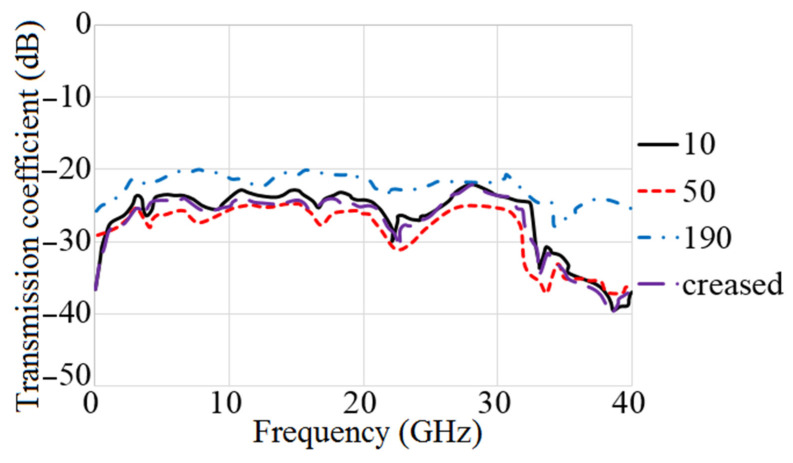
Transmission coefficient results of flexibility investigation for the proposed 8 ports UWB-MIMO (in degrees) when antenna 1 sends.

Figure 26 shows the transmission coefficient results of the antenna considering four different angles of bending. It demonstrates that the isolation of the antenna (shown by the S21 result) was improved by a smaller radius of bending until 50° due to the separation between radiating elements that get bigger as the degree bend increases. After it exceeded from 50° its level increasing towards the positives levels and mutual coupling reduces. 

## 4. Proposed MIMO Antenna for Body Contacts and Specific Absorption Rate

Another important element to consider is the Specific Absorption Rate (SAR), which indicates how much power is absorbed by human tissues and should be considered when a MIMO antenna is assumed in a close proximity to the human body, such as in smartphones and wearable devices [74]. The SAR is the amount of energy absorbed by a unit mass of tissue. The American and European standards are the two most important. The first requires a SAR value of less than 2 W/kg for every 10 g of tissue, while the second demands a SAR value of less than 1.6 W/kg for every 1 g of tissue [76].

**Figure 27:**
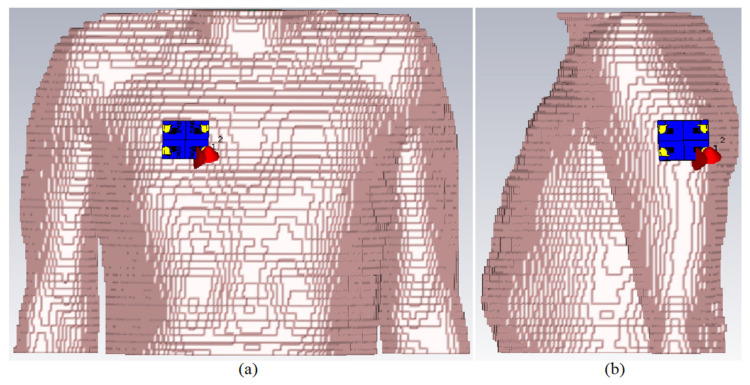
The proposed 8 ports MIMO antenna on the Gustav voxel model: (**a**) chest and (**b**) arm.

Figure 27 shows the MIMO antenna simulation setup on the arm and chest for simulation and computation of the SAR distribution owing to an 8 element MIMO antenna. At the whole working BW, computation was carried out for 1 g and 10 g tissues. The Gustav phantom body model was utilized in CST MWS. The skin, fat, muscle, and bone make up is of Gustav’s body model. Table 6 shows the average tissue characteristics for each of these bodily sections at 2.4 GHz [77]. 

**Figure 28:**
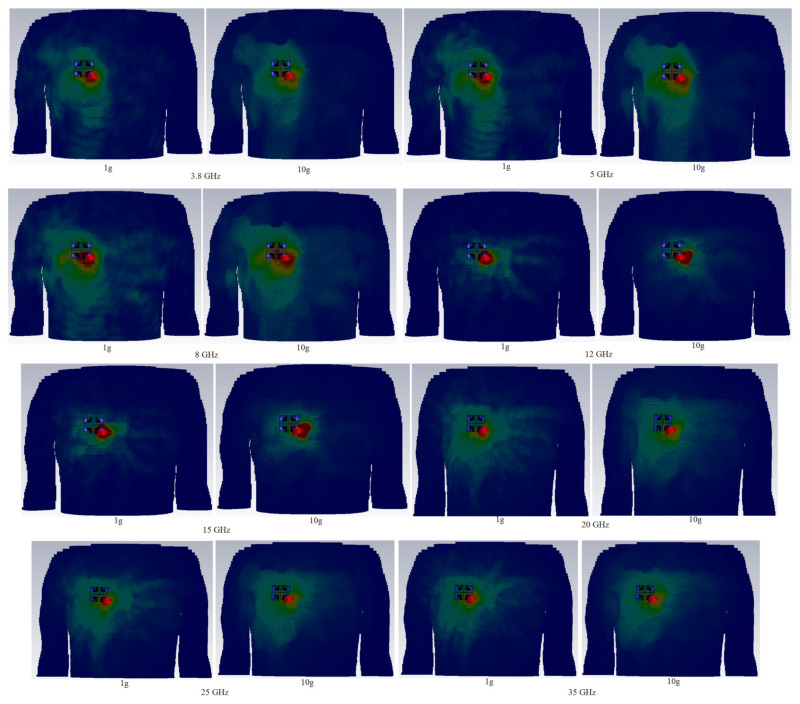
Simulated SAR distribution for the MIMO antenna on chest at different frequencies.

**Figure 29:**
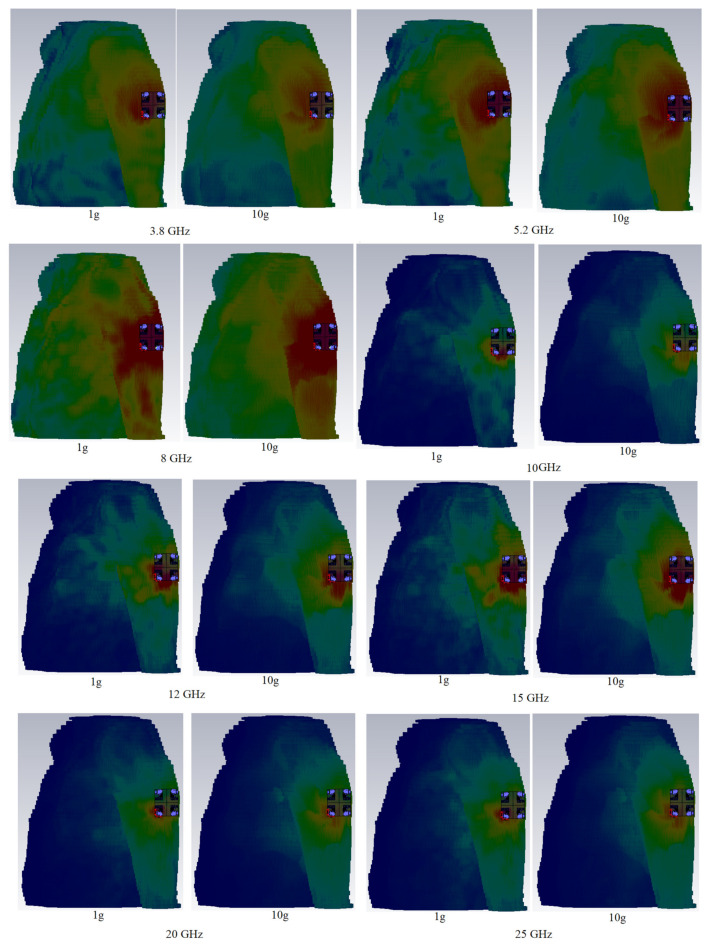
Simulated SAR distribution for the MIMO antenna on arm at different frequencies.

Table 7 displays the full SAR values for both standards. In the close proximity of the human hand and chest models, the wearable UWB-MIMO is being investigated. The suggested antenna elements’ input power at operational frequencies is expected to be 24 dBm for each element. Figure 28 and Figure 29 depict simulated SAR values that are acceptable at the required frequencies. The antenna has a 5 mm proximity and a 65° inclination angle. Table 7 indicates that the SAR values on arm, depending on 1 g and 10 g, are within acceptable ranges (port 1 is active here). It is noticed that the SAR value exceeds the standard values by increasing in frequencies (higher frequencies). Under many exposure conditions for instances in eye exposure, specifically at higher frequencies, volume averaged SAR decreases [78]. Besides, it has been illustrated that the electromagnetic radiation is absorbed more with increases in frequency [79].

The comparisons between the proposed MIMO antenna and several different works are shown in Table 8. It shows that the proposed UWB-MIMO antenna offers improved performing assuming mutual coupling, gain, operating frequency bandwidth, radiation efficiency, and the ECC. The proposed flexible 8-ports MIMO antenna presents low mutual coupling among its ports, high gain and efficiency, and low ECC. 

**Figure 30:**
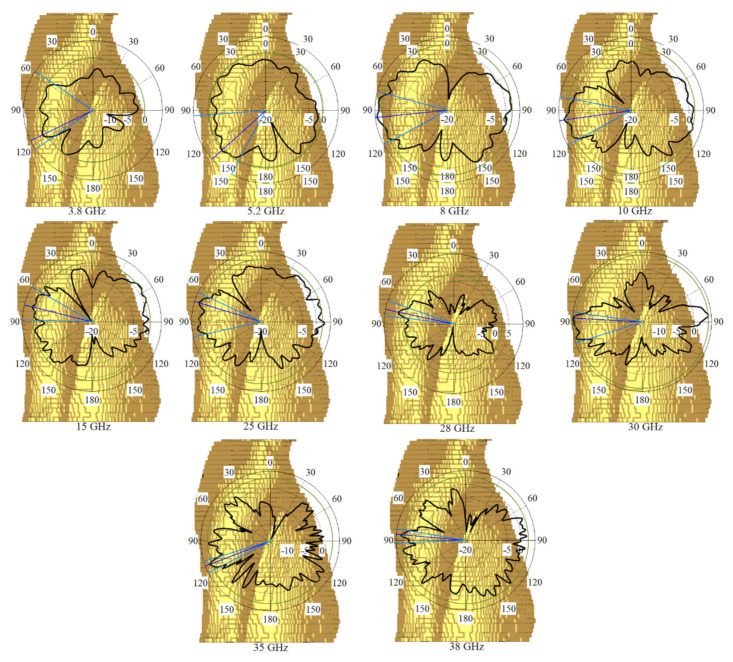
The simulated radiation pattern of the proposed MIMO antenna on arm of voxel body at different frequencies.

**Figure 31:**
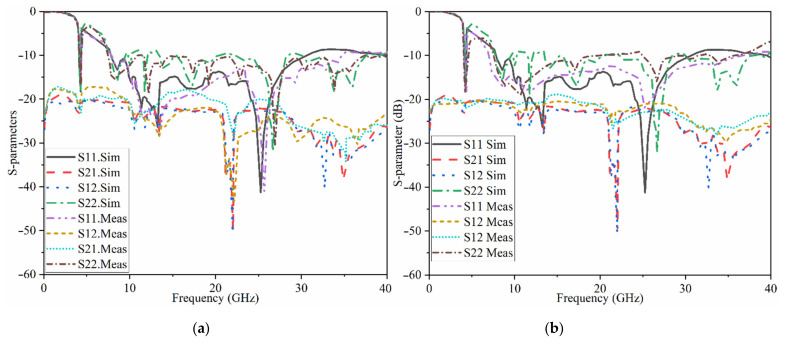
The simulated and measured reflection and transmission coefficient results on (**a**) arm and (**b**) chest for single element MIMO.

More simulations were run to see how well the suggested MIMO antenna worked when held close to the CST MWS human arm and chest voxel model. When port 2 is operational, Figure 30 depicts the antenna patterns throughout the complete resonance spectrum. The voxel model had a greater impact on vertical polarization patterns than horizontal polarization patterns because the phone primarily contacted the head and hand by its length in the presence of the voxel body. Figure 31 exhibits greater than 22 dB isolation. But there is a minor frequency shift observed when all resonances have been accomplished. Reasonably, the coupling effects from the body might influence the antenna’s characteristics that shifts its frequency. In fact, the body has higher dielectric constant compared to the simulation conducted in the vacuum.

## 5. Conclusions

A UWB-MIMO antenna based on MTM was assembled for research application within 5G, X-band, Ka and Ku bands, and satellite communications. There are four antenna components were positioned at each corner of a board, respectively, for the communication linkages. The MAVA is used in the proposed metamaterial-integrated MIMO antenna to achieve dual-polarization and polarization diversity. The antenna was developed on a denim substrate with $\varepsilon_{r}$ = 1.4, tan = 0.02 and h = 0.5 mm thickness. The suggested antenna’s dual-polarization capability is designed to decrease the significant attenuation that happens in 5G communication networks (particularly at higher frequencies) and to achieve large data speeds. Moreover, orthogonal polarization between each pair of antenna ports is used to achieve excellent isolation between antenna ports. At 3.7–3.85 GHz and 5–40 GHz, the proposed UWB-MIMO antenna has an impedance matching bandwidth. Specifically, the antenna is purposely loaded with staircase meander line, then coupled with the MTM structure in order to reduce mutual coupling effects, thus, increase its gain and efficiency. The studied UWB-MIMO antenna has a high isolation of more than 22 dB, an ECC of less than 0.01, and acceptable diversity gain. Subsequently, the Gustav voxel arm and chest were evaluated, and the SAR values at the whole working BW were thoroughly analyzed in the simulation.

In conclusion, the designed UWB-MIMO antenna gained higher isolation, efficiency, and gain; smaller footprint and size, easier implementation, and lower cross-polarization. It demonstrates that it has strongly fulfills requirements to opt functioning whether for 5G, LTE, X-band, Ka and Ku bands, or/and satellite communications. Actually, there are several problems may occur during fabricating 5G devices and antennas processes utilizing normal non-flexible board, particularly, for high frequency and mm-wave band antennas especially when incorporating them with radio frequency circuits in wearable devices and systems. We offered a compact and flexible UWB-MIMO antenna, which is suitably wearable for ultra-thin 5G mobile devices without significantly compromising the antenna’s functionability. It is open for additional functions to be incorporated into customizable designs imaginable along the future direction in its technological advancement specifically utilizing 5G and satellite communication applications.

## Figures and Tables

**Table 1:** Optimized dimensions of MAVA.

Parameter	Values (mm)	Parameter	Values (mm)
$L_{s}$	15.00	$L_{p1}$	10.00
$L_{f}$	8.60	$L_{p}$	5.40
$L_{g}$	3.80	$W_{p}$	6.00
$W_{g}$	6.50	$W_{s}$	15.00
$W_{1}$	1.40	$W_{p1}$	5.50
$r_{1}$	3.60	$L_{g1}$	1.70
$r_{2}$	4.70	$r_{3}$	4.70
$W_{g1}$	8.00	$r_{4}$	2.00
$L_{f1}$	4.65	$L_{1}$	1.25
$W_{f1}$	0.75	$W_{2}$	1.25
$L_{2}$	5.00	$L_{3}$	0.25
$L_{4}$	7.50	$L_{5}$	9.00

**Table 2:** MTM structure dimensions.

Parameters	Dimensions (mm)
$m_{1}$	4.50
$m_{l}$	1.50
$m_{W}$	0.27
S	0.50
$a_{m2}$	1.5
$a_{m1}$	1.15
$b_{m2}$	1.8
$b_{m1}$	1.4

**Table 3:** The characteristics of the MTM structure.

Parameters	$\varepsilon_{r}$	$\mu_{r}$
Operating BW (GHz)	3.2–9, 9.6–13, 13.2, 14–17.5, 19–26.5, 27.5–30	3.1–3.3, 7–10, 12.5–13.5, 13.8–14, 17.5–18.5, 23.5–27.5

**Table 4:** The performance of MTM- band MAVA with 8-ports at the resonance frequencies when ports 1 and 2 are active (Sim: Simulation, Meas: Measurement).

$f_{r}\;(\mathrm{GHz})$	Gain (dBi) Port 1 Sim/Meas	Gain (dBi) Port 2 Sim/Meas	Eff (%) Port 1 Sim	Eff (%) Port 2 Sim
3.8	4.70/3.50	10.7/10.00	93.42	97.00
5.2	3.20/2.80	4.03/3.00	97.58	98.19
8.0	4.75/3.50	4.83/4.20	95.00	96.50
10	4.10/3.90	4.88/4.50	93.42	92.00
15	4.90/3.85	4.93/4.40	88.58	94.19
20	7.25/6.90	9.5/6.85	97.42	98.00
25	5.70/5.45	7.25/5.95	93.58	98.19
29	5.40/5.00	5.78/5.20	91.50	98.00
31	5.90/5.25	5.95/5.15	95.58	97.00
34	5.70/4.95	5.90/5.01	91.50	98.00
38	4.70/4.25	6.08/5.15	95.58	97.00

**Table 5:** The simulated GD values for between Port 1 and other ports at different frequencies.

Ports/Frequency (GHz)	3.8	5.2	8	10	15	18	22	28	32	35	38
**P12**	9.30	9.98	9.95	9.98	9.86	9.99	9.99	9.99	9.99	9.99	9.98
**P13**	9.95	9.99	9.99	9.99	10	10	10	10	10	9.95	9.98
**P14**	9.94	9.99	9.99	9.99	9.99	9.99	9.98	10	9.99	9.98	10
**P15**	9.99	9.99	9.99	9.99	9.99	9.99	9.99	9.99	9.98	10	9.99
**P16**	9.94	9.88	9.99	9.99	9.99	10	10	10	9.99	10	9.98
**P17**	9.94	9.97	9.99	10	9.98	9.99	9.99	10	9.99	9.94	9.94
**P18**	9.89	9.99	9.98	9.99	9.99	10	10	9.98	9.99	9.97	9.97

**Table 6:** The average tissue characteristics of human body parts at 2.4 GHz [77].

Tissues/Properties	$\varepsilon_{r}$	σ (S/m)	*ρ* (Kg/m^3^) pls Define *ρ*
Skin	38.01	1.464	1000
Fat	5.28	0.105	1000
Muscle	52.73	1.739	1000
Bone	11.38	0.394	1000

**Table 7:** SAR values (W/kg) on arm at different frequencies.

Port 1/*f_r_*	3.8 GHz	5.2 GHz	5.4 GHz	8 GHz	10 GHz	15 GHz	20 GHz	22 GHz
SAR 1g	0.069	0.218	0.601	0.885	0.904	1.302	1.294	1.315
SAR 10g	0.028	0.087	0.440	0.619	0.816	1.115	1.108	1.128
**Port 1/*f_r_***	**25 GHz**	**28 GHz**	**30 GHz**	**32 GHz**	**35 GHz**	**38 GHz**		
SAR 1g	1.350	1.498	1.797	1.912	2.105	2.253		
SAR 10g	1.133	1.180	1.562	1.802	2.001	2.126		

**Table 8:** Comparison of proposed 8-ports MIMO antenna with several reference antennas for 5G and satellite communications.

Reference Number	Dimensions (mm)	Isolation (dB)	Max Gain (dBi)	BW (GHz)	Efficiency (%)	Max ECC
[15]	13 × 25	>20	4.80	2–12	N/A	0.009
[31]	50 × 50	>17	5.80	2–12	86.00	0.45
[24]	34 × 34	>25	6.00	2.2–11	75.00	<0.01
[23]	38.2 × 26.6	>16	N/A	2.72–12	N/A	N/A
[80]	54 × 54	>15	2.83	2–11	85.00	<0.02
[81]	40 × 20	>20	>3.00	2.5–11	N/A	<0.1
[82]	16 × 26	>22	6.86	2.82–14.45	91.70	<0.08
[83]	24 × 30	>16.3	4.80	3–12.6	N/A	<0.05
[84]	50 × 35	>21	7.48	1.83–13.82	84.00	<0.059
[85]	45 × 45	>15	7.50	21–30	78.00	<0.03
[86]	150 × 75	>12	6.20	3.3–7.1	>48	<0.07
Proposed	50 × 45	>22	10.70	3.70–3.85, 5–40	98	<0.01

## Data Availability

Not applicable.
